# Delineation of anthracyclines and herceptin induced cardiotoxicity using contrast enhanced CMR

**DOI:** 10.1186/1532-429X-16-S1-P278

**Published:** 2014-01-16

**Authors:** Yasmin S Hamirani, Ronny Jiji, Michael Salerno, Andrew Wong, Adrian Loffler, Christiana M Brenin, Patrick Dillon, Jennifer Kay, John M Christopher, Frederick H Epstein, Christopher M Kramer

**Affiliations:** 1 Cardiology, University of Virginia, Charlottesville, Virginia, USA; 2 Department of Radiology, University of Virginia, Charlottesville, Virginia, USA; 3 Department of Biomedical Engineering, University of Virginia, Charlottesville, Virginia, USA; 4 Department of Medicine, University of Virginia, Charlottesville, Virginia, USA; 5 Department of Medical Oncology, University of Virginia, Charlottesville, Virginia, USA; 6 Billings Clinic, Billings, Montana, USA

## Background

Anthracyclines and Herceptin are well known amongst chemotherapeutic agents to cause cardiotoxicity. Anthracyclines, particularly in combination with, Herceptin are seen in studies to cause cumulative, dose-related, progressive myocardial damage with decrease in left ventricular(LV) ejection fraction (EF) ultimately leading to clinical heart failure, which can be irreversible. There is lack of appropriate guidelines to monitor cardiac function in these patients. We hypothesized that changes in tissue distribution of gadolinium (Gd) and/or myocardial strain identified using (Gd)-enhanced cardiac magnetic resonance (CMR) could help identify early myocardial damage which can lead to systolic dysfunction.

## Methods

Patients naïve to chemotherapy, with newly diagnosed cancer for which athracycline or Herceptin based regimen was used, and who had normal baseline LV systolic function were recruited in the study. CMR was conducted at three time points: before chemotherapy, 20 days after completion of first round and 1 to 6 months after completion of all chemotherapy treatment. CMR measures of LV systolic and diastolic function, strain (cine dense imaging), T2 mapping, VENC (velocity encoded) imaging, and distribution of gadolinium (Gd) contrast via T1 mapping and late gadolinium enhancement were examined. High sensitive troponins were measured at the time of the CMRs.

## Results

We present data on our initial 28 patients. The mean age of our patient population is 48 ± 11 years, 86% are females, 24 have breast cancer while 2, 1 and 1 respectively have leukemia, lymphoma and sarcoma. Twenty one were treated with anthracyclines and 7 were on Herceptin. None of the patients had combined therapy. 24 patients have completed their 1st post-chemotherapy CMR, while 13(46%) have completed all three. The mean anthracycline dose was 174 ± 189 mg. LV EF drop of < 50% was noted only in 1 patient (3.5%). However diastolic dysfunction based on mitral E/A ratio(< 1 or > 2) was detected in 33% of the patients. In patients with at least one follow up CMR study, no significant trends in LV volumes, strain values or changes in the volume of distribution of gadolinium were observed (Table 1). No LGE was noted in any of the patients post-chemotherapy.

**Table 1:** 

	Baseline (n = 24)	Post-chemo study 1 (n = 24)	Post-chemo study 2 (n = 13)	p
Ejection fraction (% ) (mean ± SD)	64 ± 6.5	63 ± 8	64 ± 7	NS

Indexed LVEDV (ml/m2)	68.3 ± 15	65 ± 17	69 ± 13	NS

Indexed LVESV (ml/m2)	25 ± 8	25 ± 9.13	25 ± 7	NS

Pre-Contrast T1 (ms)	976 ± 31	971 ± 36	984 ± 34	NS

Partition co-efficient (Λ) (0,5,15, 20 min)	0.43 ± 0.30	0.41 ± 0.37	0.44 ± 0.36	NS

Partition co-efficient (Λ) (0, 10, 15, 20 min)	0.42 ± 0.02	0.41 ± 0.03	0.44 ± 0.03	NS

Volume of distribution (Vd) (0,5,10,15,20)	0.27 ± 0.02	0.27 ± 0.03	0.28 ± 0.02	NS

Average peak systolic radial strain (Err) (ms)	0.17 ± 0.02	0.17 ± 0.02	0.16 ± 0.03	NS

Average peak systolic circumferential strain (Ecc) (ms)	-0.17 ± 0.03	-0.17 ± 0.03	-0.18 ± 0.04	NS

Average systolic strain rate (SSR) (ms)	1.07 ± 0.11	1.06 ± 0.2	1.14 ± 0.33	NS

Average diastolic strain rate (DSR) (ms)	0.96 ± 0.19	0.95 ± 0.27	0.90 ± 0.49	NS

## Conclusions

Except one patient, no significant (< 50%) drop in left ventricular ejection fraction was seen in our patients treated with Anthracyclines or Herceptin. Thus, we were unable to show a benefit of CMR in detecting predisposing factors to an EF decline. However with further follow-up, additional patients may demonstrate a decline. The reduced incidence of LV dysfunction compared to historical incidence may be due to a recent shift in oncology practice with regards to the use of these chemotherapeutic agents, including avoiding simultaneous use of Anthracyclines with Herceptin and reduction in the maximum Anthracyclines dosage.

## Funding

AHA (American heart association).

